# Does Smartphone Addiction, Social Media Addiction, and/or Internet Game Addiction Affect Adolescents’ Interpersonal Interactions?

**DOI:** 10.3390/healthcare10050963

**Published:** 2022-05-23

**Authors:** Shang-Yu Yang, Yu-Chi Wang, Ya-Chen Lee, Ying-Lien Lin, Pei-Lun Hsieh, Pin-Hsuan Lin

**Affiliations:** 1 Department of Healthcare Administration, College of Medical and Health Science, Asia University, Taichung 41354, Taiwan; henry879019@yahoo.com.tw; 2 Department of Chinese Medicine, China Medical University Hospital, Taichung 40402, Taiwan; cuycuy24@gmail.com; 3 Department of Occupational Therapy, College of Medical and Health Science, Asia University, Taichung 41354, Taiwan; jennyleenet@asia.edu.tw; 4 Department of Industrial and Information Management, National Cheng Kung University, Tainan City 71101, Taiwan; r38021019@gs.ncku.edu.tw; 5 Department of Nursing, College of Health, National Taichung University of Science and Technology, Taichung 40401, Taiwan; peilun@nutc.edu.tw; 6 Department of Health and Beauty, Shu Zen Junior College of Medicine and Management, Kaohsiung 82144, Taiwan

**Keywords:** smartphone addiction, social media addiction, internet game addiction, interpersonal interactions

## Abstract

The purpose of this study is to investigate the correlations that levels of addiction to smartphones, social media, and online games have with levels of real-life and online interpersonal interactions among adolescents. In this cross-sectional study of adolescents in a college in Taiwan, structured questionnaire surveys were used to collect information. The questionnaire included the following: demographic background, Real Interpersonal Interaction Scale (RIIS), Internet Interpersonal Interaction Scale (IIIS), Smartphone Application-Based Addiction Scale (SABAS), Bergen Social Media Addiction Scale (BSMAS), and Internet Gaming Disorder Scale—Short Form (IGDS9-SF). Multiple regression analyses were carried out to investigate the correlations between SABAS, BSMAS, IGDS9-SF, and RIIS/IIIS. We recruited 998 students (413 boys). The average age was 17.18 ± 1.46. The study results show that adolescents with higher levels of addiction to smartphones and social media may have greater interpersonal interaction with friends in real life, but adolescents with high levels of addiction to online games may have less interpersonal interactions with friends in real life. Adolescents with high levels of addiction to smartphones, social media, and online games may have greater interpersonal interactions with friends online.

## 1. Introduction

Interpersonal interactions play a critical role in the physical and mental health of adolescents. In the process of growing up, the interactions that adolescents have with parents since childhood play a key role in their physical and mental health [1,2]. Adolescents with amicable interactions with parents develop healthier personal character traits, such as warm and enthusiastic personalities [3] and a higher likelihood of good academic achievement [4,5]. Conversely, the absence of amicable interactions may increase the risks of emotional disorders [6,7] and even mental illness [8]. Furthermore, during adolescence, peers play a crucial role in affecting adolescent emotions, social interactions, health behavior, and decision-making [9,10]. Adolescents build self-identity, self-esteem, and self-efficacy through interactions with peers [9,11] and, in the process, learn stress resistance [12]. As a result, the investigation and understanding of the factors influencing adolescent interpersonal interactions are crucial.

Adolescent interpersonal interactions are affected by many factors. Previous research has shown that gender, obesity, religion, and addiction to tech products can affect interpersonal interactions [13,14,15]. The popularity and multifunctionality of tech products, such as smartphones, have led to an increase in screen time and a decrease in real-life interpersonal interactions among adolescents due to their intemperate use of tech products [15]. One study reported that adolescents who spend more time staring at screens spend less time on non-screen-related activities, such as face-to-face social interactions, sports, or religious activities [16]. Smartphones are the most commonly used tech product among adolescents nowadays, primarily for the purpose of social media and online gaming [17,18]. In Taiwan, young people spend more than five hours a week using smartphones, more than three hours on social media, and more than 1.5 h on online gaming [19].

The use of social media (such as Facebook, Instagram, and Twitter) is a highly popular activity among adolescents; however, such use also increases the risks of social media addiction [17]. Additionally, an analogous phenomenon occurs in online games [20]. Especially in Taiwan, nearly one-fifth of high school students have experienced internet addiction, and men seem to be more likely to have internet addiction [21,22]. Although previous studies have shown that the improper use of addiction to electronics may affect the interpersonal interactions of adolescents (i.e., parent-child interactions), no conclusive findings have been furnished on whether interpersonal interactions are increased or decreased by such use [23,24,25]. The absence of conclusive findings is attributable to previous studies not differentiating between the locations—particularly, real-life or (with online-only acquaintances) non-real-life—in which interpersonal interactions occur. Moreover, previous studies failed to clarify who (particularly peers vs. parents) is influencing interpersonal interactions.

In Taiwan, approximately a quarter (24.9%) of adolescents are predisposed to smartphone addiction [26]. Adolescents addicted to smartphones spend most of their time on social networking sites, followed by games, messaging, taking photos, and listening to music [27,28,29]. Although smartphones were previously considered tools for maintaining interpersonal relationships or developing social networks [23], smartphone addiction may lead to alienation from and/or further deterioration of real-life interpersonal relationships [27,30]. Furthermore, the use of phone apps is the key factor in the forming of smartphone addiction in adolescents [26]. Thus, in researching the impact of technological products on interpersonal interactions among adolescents, it is necessary not only to investigate the impact of tech hardware such as smartphones but also further explore the impact of tech software (applications). Additionally, according to the situation where interpersonal interaction occurs (e.g., real-life vs. online), we can fully understand the impact of interpersonal interaction on the addiction to a smartphone and its applications (such as social media or games).

The purpose of this study was primarily to explore the correlations that levels of addiction to smartphones, social media, and online games have with levels of real-life and online interpersonal relationships among adolescents and further distinguish the relative influence that these factors have on interpersonal interactions in real life.

## 2. Materials and Methods

### 2.1. Study Participants

A cross-sectional study with participants from a junior college of medical care in Kaohsiung city (southern Taiwan) was conducted. Students enter the school through a written review and a selective admissions process. First, the research assistants went to each class for recruitment (and posted recruitment posters). Research assistants gave a full explanation in class to all students, and all eligible participants were fully aware of their right to withdraw from participation anytime during the study period. Then, after obtaining written consent from the participants (for students younger than 18 years, consent from their guardian was required), data were collected using structured questionnaire surveys, and 1001 surveys were collected. Three questionnaires were found to be invalid due to incomplete questionnaires, leaving 998 valid questionnaires (completed questionnaires) for analysis. The inclusion criteria for participant recruitment were 1. owning a smartphone for over a year and 2. being able to communicate in Mandarin and answer the questionnaire in written Chinese. Incomplete questionnaires were excluded. The questionnaires were collected over three months, from May 2019 to July 2019. Ethical approval for the study was obtained from the National Cheng Kung University Human Research Ethics Committee (NCKU HREC-E-108-032-2). Finally, we adopted 998 validated responses students (413 boys), and the average age was 17.18 ± 1.46.

### 2.2. Questionnaire

The questionnaire (online questionnaire) comprised six portions. The first portion covered participants’ demographic information, such as sex, age, body mass index, religion, exercise (specifically, the number of days per week the participant engaged in exercise for 30 min or more), monthly allowance, partnership, and type of current residence (home, dorm, or off-campus housing). The second portion was the Real Interpersonal Interaction Scale (RIIS), a 14-item self-evaluation scale formulated by Chen [31] that measures the level of interaction in real-life interpersonal relationships (i.e., interactions where one meets with the other in person). The RIIS has three subscales. The first subscale, *intimacy with parents*, comprises six statements (e.g., “I share my secrets with my parents”); higher scores signify more intimate conversations with parents. The second subscale, *intimacy with friends*, has four statements (e.g., “I discuss my feelings with my classmates or friends”); higher scores signify more intimate conversations with friends. The third subscale, *informational disclosure with friends*, has four statements (e.g., “I talk about my school life or schoolwork with my classmates or friends”); higher scores signify greater disclosure of information by the participant to their friends. Each statement was graded on a four-point Likert scale, with 1 representing *never* and 4 representing *often*. After tallying the points for each statement, a higher total score signified more intimate real-life interpersonal relationships. RIIS was shown to be valid and reliable [31]. In this study, the Cronbach alpha was 0.93 for the RIIS total score and 0.84–0.92 for the three subscales.

The third portion of the questionnaire is the Internet Interpersonal Interaction Scale (IIIS). This scale, like the RIIS, was formulated by Chen [31] and measured the level of interaction in online interpersonal relationships (i.e., acquaintances that a person has never met in person and only interacts with online) using 10 self-evaluation statements. IIIS is divided into two subscales. *Intimacy with online friends* has six statements (e.g., “I talk about my feelings with online friends”); higher scores signified more intimate discussions with online friends. *Informational disclosure with online friends* has four statements (e.g., “I talk about school life or schoolwork with online friends”); higher scores signified greater disclosure of information by the participant to their online friends. Each statement was graded on a four-point Likert scale, with 1 representing *never* and 4 representing *often*. After tallying the points for each statement, a higher total score signified more intimate interpersonal relationships. IIIS was shown to be valid and reliable (Chen, 2002). In this study, the Cronbach alpha was 0.97 for the IIIS total score and 0.95 and 0.92 for the two subscales.

The fourth portion is a Chinese version of the Smartphone Application-Based Addiction Scale (SABAS), which uses six self-evaluation statements to measure the risk of smartphone addiction; for example, one such statement is “My smartphone is the most important thing in my life” [32,33]. Each statement was graded on a six-point Likert scale, with 1 representing *strongly disagree* and 6 representing *strongly agree*. Higher scores signified higher levels of smartphone addiction. The Chinese version of the SABAS was shown to be valid and reliable [19,32]. In this study, the Cronbach alpha was 0.81 for the SABAS total score.

The fifth portion is a Chinese version of the Bergen Social Media Addiction Scale (BSMAS). This scale was adapted from the Bergen Facebook Addiction Scale by Griffiths and used six self-evaluation statements to measure levels of addiction to social media platforms—such as Facebook, Twitter, and Instagram—within the past year [34,35,36]. As an example, one such statement is, “You spend a lot of time thinking about social media or on planning how to use it”. Each statement was graded on a five-point Likert scale, with 1 representing *very rarely* and 5 representing *very often*. Higher scores signified higher levels of addiction to social media. The Chinese version of BSMAS was shown to be valid and reliable [19,32,37,38]. In this study, the Cronbach alpha was 0.87 for the BSMAS total score.

The sixth portion of the questionnaire is a Chinese version of the Internet Gaming Disorder Scale—Short Form (IGDS9-SF). This scale uses nine self-evaluation statements to measure Internet Gaming Disorder for the previous year [32,39]. As an example, one such statement is “Do you systematically fail when trying to control or stop your gaming activity?” Each statement was graded on a five-point Likert scale, with 1 representing *never* and 5 representing *very often*. Higher scores signified higher levels of problematic gaming. The Chinese version of the IGDS9-SF was shown to be valid and reliable [19,32,37,40,41]. In this study, the Cronbach alpha was 0.94 for the IGDS9-SF total score.

### 2.3. Statistical Analysis

In this study, data analysis was conducted using SPSS 22.0 for Mac (IBM Corp., Armonk, NY, USA). First, descriptive statistics were presented for the demographic data collected from the questionnaires. Subsequently, the correlations between RIIS, IIIS, SABAS, BSMAS, and IGDS9-SF were analyzed using Pearson correlation coefficient analysis. Finally, multiple linear regression analysis was used to verify the correlations among RIIS, IIIS, SABAS, BSMAS, and IGDS9-SF. The total score and subscale scores for both RIIS and IIIS were constructed into the multiple regression model as a dependent variable, and the total scores for BSMAS, SABAS, and IGDS9-SF were constructed, respectively, as an independent variable; all demographic variables were simultaneously adjusted in the regression models. Significance was indicated by a *p*-value of 0.0024 (0.05/21) for multiple linear regression analysis (0.05 for Pearson correlation coefficient analysis), derived after the adoption of Bonferroni Adjustments.

## 3. Results

### 3.1. Participant Demography

Participant demographics are presented in Table 1. The age range of participants was 14–20 years, with an average age of 17.14 years (SD = 1.33). More than half of the participants exercised two days or more each week (56.6%), and more than half had a weekly allowance of less than TWD 4000 (54.8%). Over three-quarters of participants are religious (78.1%), and over three-quarters live at home (77.2%).

### 3.2. Pearson Correlation Coefficient Analysis

Results for the Pearson correlation coefficient analysis of the correlation among RIIS, IIIS, SABAS, BSMAS, and IGDS9-SF are presented in Table 2. For the RIIS, a significant correlation (r = −0.12 to −0.15, *p* < 0.05) was noted among SABAS, BASMAS, and the two RIIS subscales (*intimacy with friends* and *informational disclosure with friends*); significantly negative correlations between IGDS9-SF and the RIIS total and subscale scores (r = −0.07 to −0.15, *p* < 0.05) were also observed. For the IIIS portion, all three SABAS, BSMAS, and IGDS9-SF scores exhibited significantly positive correlations with the IIIS total and subscale (*intimacy with friends* and *informational disclosure with friends*) scores (r = 0.19 to 0.28, *p* < 0.01).

### 3.3. Multiple Linear Regression Analysis

Multiple regression analyses for identifying RIIS/IIIS with their dimensions significantly related to SABAS are presented in Table 3. A significantly positive correlation (β = 0.11~0.14, *p* < 0.0024) was noted between the SABAS and the RIIS subscales, *intimacy with friends* and *informational disclosure with friends*. This result indicates that higher levels of smartphone addiction entail higher levels of intimacy with friends and disclosure of information to friends in real life. Furthermore, a significantly positive correlation (β = 0.19~0.23, *p* < 0.0024) was noted between SABAS and the IIIS total score, *intimacy with online friends* and *informational disclosure with online friends*. This result indicates that higher levels of smartphone addiction entail higher levels of online interpersonal interaction, intimacy with online friends, and informational disclosure with online friends.

Multiple regression analyses for identifying RIIS/IIIS with their dimensions significantly related to BSMAS are presented in Table 4. BSMAS was also significantly and positively correlated with the RIIS subscale *intimacy with friends* (β = 0.11, *p* < 0.0024). This result indicates that higher levels of social media addiction entail higher levels of intimacy with friends in real life. BSMAS and the IIIS total score, *intimacy with online friends* and *informational disclosure with online friends* were also significantly and positively correlated (β = 0.23~0.25, *p* < 0.0024). This result indicates that higher levels of social media addiction entail higher levels of online interpersonal interaction, intimacy with online friends, and informational disclosure with online friends.

Multiple regression analyses for identifying RIIS/IIIS with their dimensions significantly related to IGDS9-SF are presented in Table 5. IGDS9-SF and the RIIS subscales *intimacy with friends* and *informational disclosure with friends* were significantly and negatively correlated (β = −0.12~−0.14, *p* < 0.0024). This result indicates that higher levels of online gaming addiction entail lower levels of intimacy with friends and disclosure of information to friends in real life. Furthermore, IGDS9-SF and the IIIS total score, *intimacy with online friends* and *informational disclosure with online friends* were significantly and positively correlated (β = 0.26~0.27, *p* < 0.0024). This result indicates that higher levels of online gaming addiction entail higher levels of online interpersonal interactions, intimacy with online friends, and informational disclosure with online friends.

## 4. Discussion

Relative to the literature, this study is a more complete investigation into the relationship between tech addiction, including generalized (SABAS) and specific internet addiction (BSMAS and IGDS9-SF [19,37,42,43,44,45], and interpersonal interactions among adolescents. This study shows that higher levels of smartphone and social media addiction might not decrease interpersonal interactions with friends in real life, but high levels of online gaming addiction might lower interpersonal interactions with friends in real life; high levels of a smartphone, social media, and online gaming addiction might increase interpersonal interactions with online friends.

Our results showed that higher levels of smartphone addiction were correlated with higher levels of intimacy with friends and disclosure of information both online and in real life. This suggests that adolescents may not significantly decrease their interpersonal interactions in real life despite their prolonged use of smartphones. This finding is inconsistent with that of Twenge et al. (2018), who studied US adolescents from 1991 to 2016. Specifically, they argued that greater time spent on electronic communications and on screens results in lesser time spent on non-screen-related activities, such as face-to-face social interactions. This inconsistency might be attributable to the popularity of smartphones among adolescents in recent years, particularly the widespread use of smartphones for social media among adolescents [46]. Additionally, the RIIS total score and the *intimacy with parents* subscale score were not significantly correlated, suggesting that after obtaining a cell phone, adolescents only increased interactions with friends through their phones [29] but did not significantly increase emotional interactions with their parents. This result also reflects the wider phenomenon that interpersonal interactions during adolescence primarily occur with peers. Specifically, during adolescence, a person gradually expands their horizons and circle of friends, in addition to developing their personal and social identity through diverse interpersonal interactions (such as through making friends outside their school) [47]. It is also important for parents to understand how teens behave like this during this period.

Further analysis demonstrates that SABAS had a stronger association with IIIS (β = 0.19–0.23) than with RIIS (β = 0.11–0.14). This result suggests that adolescent addiction to smartphones is partly due to the enjoyment derived from interaction with online friends that they have never met in person (as defined in this study). An analysis of BSMAS correlations also yielded results similar to those for SABAS. A recent study [48] argued that adolescents with phone addiction might experience psychological issues—such as low self-esteem and aggressive behavior—that may negatively affect their real-life interactions with peers; this may also be a factor in driving phone addicts toward social interactions online. Moreover, although smartphones may assist adolescents in socializing, smartphone overuse may cause biological or psychological problems, such as poor sleep quality, musculoskeletal discomfort, and dampened spirits [28,49,50,51,52]. In addition, disputes over money, verbal violence, and cyberbullying may occur on the internet; parents and other stakeholders should pay special attention to these issues.

Higher levels of social media addiction were correlated with higher levels of intimacy with friends in real life, which suggests that social media affects real-life interpersonal interactions, particularly in bringing friends closer. However, social media use was not associated with intimacy with parents. This may suggest that adolescents are more predisposed toward interacting and sharing their feelings with their peers and less predisposed to sharing with their parents or elders; some adolescents may even refuse to add their parents to their social media [53]. Adolescents use social media to integrate into peer groups, to spend time with peers, or for entertainment [54]. Conversely, higher levels of social media addiction correlate with higher levels of interpersonal interactions online (Table 3), consistent with the results for SABAS in this study. In other words, the use of social media—particularly Facebook, Twitter, and Instagram—can increase the sharing and exchange of information between users and netizens on the internet. Prior research argued that people with social media addiction are predisposed toward extraversion, neuroticism, and the fear of missing out [55]. Specifically, people with extraversion enjoy group interactions, people with neuroticism enjoy using social media to receive attention and affirmation from others, and people with a fear of missing out are worried about being ignored or excluded by the group and may therefore proactively pay attention to or participate in social activities [55].

Online game development has shifted from single-player games to multi-player games over the past few years, resulting in the creation of interactive platforms for players that also encourage online social interaction outside of gameplay [56]. In particular, players can roleplay as their game characters and temporarily escape their real-life roles (and the accompanying pressures and negative emotions endured in real life) to interact with the characters played by other players, creating more emotional exchanges and a more realistic interactive experience. This type of multi-person online interactive platform increases the motivation for users to immerse themselves in the game and remain on the gaming platform [57], where the longer a player spends on the platform, the fewer opportunities they have to experience face-to-face contact with people in real life. As shown by the results in this study, higher levels of online gaming addiction correlate with lower intimacy and informational disclosure with friends in real life and (conversely) with higher levels of intimacy and informational disclosure with online friends.

This study has several limitations. First, interpersonal interactions and addiction to tech products were measured using self-evaluation surveys. These scales in these surveys, although widely used and psychometrically reliable, cannot represent actual interpersonal interactions and addictive behaviors (or internet disorder). Second, causality could not be demonstrated through the cross-sectional research design of this study; causality in the relationship between tech-product use and interpersonal interactions requires further research. Third, this study did not measure personality traits (such as introversion or extroversion), which may cause research limitations [58,59,60]. Lastly, the participants were students from the same school, which limits the generalizability of our findings. Despite these limitations, however, the findings of this study assist parents and educational institutions in understanding the effects of tech products on adolescents.

## 5. Conclusions

This study showed that adolescents with higher levels of smartphone and social media addiction had more interpersonal interactions with friends in real life, but adolescents with higher levels of online gaming addiction had fewer interpersonal interactions with friends in real life. Adolescents with higher levels of addiction to smartphones, social media, and online gaming also had greater interpersonal interactions with online friends. Because this study was not a true study design, its findings do not allow us to conclude causality in the relationship between tech addiction and interpersonal interactions. Nonetheless, an understanding of the correlation between tech-product use and interpersonal interactions can help ameliorate the negative effects of the use of tech products. In addition, the results of this study also imply that addiction to online games may have a negative impact on real-life interpersonal interactions, which deserves the attention of parents and relevant units. On the other hand, utilizing smartphones, mobile applications, and social media may provide a more effective way of enhancing human interaction.

## Figures and Tables

**Table 1:** Background information of the participants.

	Total	Male	Female	*p*-Value
	N = 998	N = 413	N = 585	
Sex Male Female	413 (41.4%) 585 (58.6%)			
Age (mean ± SD)	16.73 ± 0.94	16.70 ± 0.84	16.75 ± 1.00	
BMI (mean ± SD)	20.77 ± 4.03	20.90 ± 3.82	20.68 ± 4.18	
Religion (n, %)				0.57
No	779 (78.1%)	326 (78.9%)	453 (77.4%)	
Yes	219 (21.9%)	87 (21.1%)	132 (22.6%)	
Exercise per week 0–1 days 2–3 days ≥4 days	433 (43.4%) 347 (34.8%) 218 (21.8%)	176 (42.6%) 126 (30.5%) 111 (26.9%)	257 (43.9%) 221 (37.8%) 107 (18.3%)	<0.01
Money can be spent each month				0.14
<4000 NTD (÷135 USD)	547 (54.8%)	215 (52.1%)	332 (56.8%)	
4000–5999 NTD (135–200 USD)	225 (22.5%)	106 (25.7%)	119 (20.3%)	
6000–7999 NTD (200–270 USD)	95 (9.5%)	43 (10.4%)	52 (8.9%)	
≥8000 NTD(≥270 USD)	131 (13.1%)	49 (11.9%)	82 (14.0%)	
Have a boy/girl friend				0.78
No Yes	696 (69.7%) 302 (30.3%)	290 (70.2%) 123 (29.8%)	406 (69.4%) 179 (30.6%)	
Living place				0.12
Home	721 (77.2%)	286 (69.2%)	435 (74.4%)	
School dormitory	117 (11.7%)	58 (14.0%)	59 (10.1%)	
Off-campus rental house	160 (16.0%)	69 (16.7%)	91 (15.6%)	
RIIS (mean ± SD)				
Total score	38.21 ± 8.90	37.33 ± 9.57	38.83 ± 8.35	0.01 *
Intimacy with parents	15.07 ± 4.76	14.92 ± 4.95	15.18 ± 4.63	0.39
Intimacy with friends	11.40 ± 2.82	11.01 ± 2.99	11.68 ± 2.67	<0.01
Informational disclosure with friends	11.74 ± 2.79	11.41 ± 2.99	11.97 ± 2.62	<0.01
IIIS (mean ± SD)				
Total score	17.14 ± 7.59	17.70 ± 7.79	16.76 ± 7.43	0.05
Intimacy with online friends	10.12 ± 4.55	10.43 ± 4.65	9.90 ± 4.48	0.07
Informational disclosure with online friends	7.03 ± 3.20	7.27 ± 3.27	6.86 ± 3.14	0.05 *
SABAS (mean ± SD)	12.14 ± 4.42	12.44 ± 4.89	11.92 ± 4.04	0.08
BSMAS (mean ± SD)	18.12 ± 5.57	18.19 ± 5.82	18.07 ± 5.39	0.74
IGD9-SF (mean ± SD)	14.77 ± 6.50	16.56 ± 7.23	13.52 ± 5.61	<0.01

BMI: Body mass index; NTD: New Taiwan Dollars; USD: United States Dollar; RIIS: Real Interpersonal Interaction Scale; IIIS: Internet Interpersonal Interaction Scale; SABAS: Smartphone Application-Based Addiction Scale; BSMAS: Bergen Social media Addiction Scale; IGD9-SF: Internet Gaming Disorder Scale. * *p* < 0.05.

**Table 2:** Correlation coefficients among Smartphone Application-Based Addiction Scale (SABAS), Bergen Social media Addiction Scale (BSMAS), Internet Gaming Disorder Scale (IGD9-SF), and Real Interpersonal Interaction Scale (RIIS)/Internet Interpersonal Interaction Scale (IIIS), including total score and subscales.

	SABAS			BSMAS			IGD9-SF		
	Total	Male	Female	Total	Male	Female	Total	Male	Female
RIIS									
Total score	0.06	0.11 *	0.01	0.05	0.08	0.04	−0.13 **	−0.07	−0.16 **
Intimacy with parents	−0.05	0.02	−0.11 **	−0.00	0.03	−0.03	−0.07 *	−0.01	−0.13 **
Intimacy with friends	0.15 **	0.17 **	0.13 **	0.13 **	0.11 *	0.11 **	−0.14 *	−0.10 *	−0.13 **
Informational disclosure with friends	0.12 **	0.16 **	0.09 *	0.07 *	0.09	0.07	−0.15 *	−0.11	−0.16 **
IIIS									
Total score	0.22 **	0.14 **	0.28 **	0.25 **	0.20 **	0.28 **	0.28 **	0.29 **	0.25 **
Intimacy with online friends	0.23 **	0.15 **	0.28 **	0.25 **	0.21 **	0.28 **	0.27 **	0.29 **	0.24 **
Informational disclosure with online friends	0.19 **	0.12 *	0.26 **	0.23 **	0.19 **	0.26 **	0.27 **	0.28 **	0.26 **

RIIS: Real Interpersonal Interaction Scale; IIIS: Internet Interpersonal Interaction Scale; SABAS: Smartphone Application-Based Addiction Scale; BSMAS: Bergen Social media Addiction Scale; IGD9-SF: Internet Gaming Disorder Scale. * *p* < 0.05, ** *p* < 0.01.

**Table 3:** Multiple regression analysis for identifying Real Interpersonal Interaction Scale (RIIS) /Internet Interpersonal Interaction Scale (IIIS) with their dimensions significantly related to Smartphone Application-Based Addiction Scale (SABAS).

	SABAS ^†^					Male ^††^					Female ^††^				
	B	SE	Beta	95% CI	*p*	B	SE	Beta	95% CI	*p*	B	SE	Beta	95% CI	*p*
RIIS															
Total score	0.09	0.05	0.06	−0.01, 0.19	0.09	0.20	0.08	0.12	0.04, 0.37	0.02 *	−0.01	0.07	−0.01	−0.14, 0.12	0.89
Intimacy with parents	−0.04	0.03	−0.04	−0.09, 0.02	0.18	0.04	0.04	0.05	−0.05, 0.12	0.36	−0.10	0.04	−0.11	−0.17, −0.03	<0.01
Intimacy with friends	0.07	0.02	0.14	0.04, 0.10	<0.01	0.09	0.03	0.17	0.04, 0.14	<0.01	0.05	0.02	0.11	0.01, 0.09	0.01 *
Informational disclosure with friends	0.06	0.02	0.11	0.03, 0.09	<0.01	0.08	0.03	0.15	0.03, 0.13	<0.01	0.04	0.02	0.07	−0.01, 0.08	0.08
IIIS															
Total score	0.30	0.04	0.22	0.21, 0.38	<0.01	0.19	0.07	0.14	0.06, 0.32	<0.01	0.38	0.06	0.28	0.27, 0.49	<0.01
Intimacy with online friends	0.19	0.03	0.23	0.14, 0.24	<0.01	0.12	0.04	0.15	0.04, 0.20	<0.01	0.24	0.03	0.29	0.18, 0.31	<0.01
Informational disclosure with online friends	0.11	0.02	0.19	0.07, 0.15	<0.01	0.07	0.03	0.12	0.02, 0.13	0.01 *	0.14	0.02	0.24	0.10, 0.19	<0.01

^†^ Controlled for sex, age, BMI, religion, exercise per week, money can be spent each month, have a boy/girl friend, and living place; ^††^ Controlled for age, BMI, religion, exercise per week, money can be spent each month, have a boy/girl friend, and living place; B: unstandardized regression coefficient; SE: standard error; CI: confidence interval; RIIS: Real Interpersonal Interaction Scale; IIIS: Internet Interpersonal Interaction Scale; SABAS: Smartphone Application-Based Addiction Scale. * *p* < 0.05.

**Table 4:** Multiple regression analysis for identifying Real Interpersonal Interaction Scale (RIIS)/Internet Interpersonal Interaction Scale (IIIS) with their dimensions significantly related to Bergen Social media Addiction Scale (BSMAS).

	BSMAS ^†^					Male ^††^					Female ^††^				
	B	SE	Beta	95% CI	*p*	B	SE	Beta	95% CI	*p*	B	SE	Beta	95% CI	*p*
RIIS															
Total score	0.11	0.07	0.06	−0.02, 0.24	0.09	0.16	0.10	0.08	−0.04, 0.36	0.12	0.07	0.09	0.04	−0.10, 0.24	0.40
Intimacy with parents	0.01	0.04	0.00	−0.07, 0.07	0.98	0.05	0.05	0.04	−0.06, 0.15	0.38	−0.03	0.05	−0.03	−0.13, 0.06	0.49
Intimacy with friends	0.07	0.02	0.11	0.03, 0.11	<0.01	0.06	0.03	0.10	0.00, 0.13	0.04 *	0.07	0.03	0.10	0.02, 0.12	0.01 *
Informational disclosure with friends	0.04	0.02	0.07	0.00, 0.08	0.03 *	0.05	0.03	0.08	−0.01, 0.11	0.12	0.04	0.03	0.06	−0.02, 0.09	0.18
IIIS															
Total score	0.42	0.05	0.24	0.31, 0.52	<0.01	0.32	0.08	0.20	0.17, 0.48	<0.01	0.50	0.07	0.27	0.36, 0.65	<0.01
Intimacy with online friends	0.25	0.03	0.25	0.19, 0.32	<0.01	0.20	0.05	0.21	0.10, 0.29	<0.01	0.31	0.04	0.27	0.22, 0.39	<0.01
Informational disclosure with online friends	0.16	0.02	0.23	0.12, 0.21	<0.01	0.13	0.03	0.19	0.06, 0.19	<0.01	0.20	0.03	0.25	0.14, 0.26	<0.01

^†^ Controlled for sex, age, BMI, religion, exercise per week, money can be spent each month, have a boy/girl friend, and living place; ^††^ Controlled for age, BMI, religion, exercise per week, money can be spent each month, have a boy/girl friend, and living place; B: unstandardized regression coefficient; SE: standard error; CI: confidence interval; RIIS: Real Interpersonal Interaction Scale; IIIS: Internet Interpersonal Interaction Scale; BSMAS: Bergen Social media Addiction Scale. * *p* < 0.05.

**Table 5:** Multiple regression analysis for identifying Real Interpersonal Interaction Scale (RIIS)/Internet Interpersonal Interaction Scale (IIIS) with their dimensions significantly related to Internet Gaming Disorder Scale (IGD9-SF).

	IGD9-SF ^†^					Male ^††^					Female ^††^				
	B	SE	Beta	95% CI	*p*	B	SE	Beta	95% CI	*p*	B	SE	Beta	95% CI	*p*
RIIS															
Total score	−0.15	0.05	−0.11	−0.24, −0.07	0.01 *	−0.09	0.07	−0.07	−0.22, 0.04	0.18	−0.22	0.06	−0.15	−0.34, −0.10	<0.01
Intimacy with parents	−0.04	0.02	−0.06	−0.09, 0.01	0.08	0.00	0.03	−0.00	−0.07, 0.07	0.99	−0.09	0.03	−0.11	−0.16, −0.02	0.01 *
Intimacy with friends	−0.05	0.01	−0.12	−0.08, −0.02	<0.01	−0.04	0.02	−0.10	−0.08, −0.00	0.04 *	−0.06	0.02	−0.12	−0.10, −0.02	<0.01
Informational disclosure with friends	−0.06	0.01	−0.14	−0.09, −0.03	<0.01	−0.05	0.02	−0.12	−0.09, −0.01	0.02 *	−0.07	0.02	−0.16	−0.11, −0.04	<0.01
IIIS															
Total score	0.32	0.04	0.27	0.24, 0.39	<0.01	0.30	0.05	0.28	0.20, 0.41	<0.01	0.34	0.05	0.25	0.23, 0.44	<0.01
Intimacy with online friends	0.18	0.02	0.26	0.14, 0.23	<0.01	0.18	0.03	0.28	0.12, 0.24	<0.01	0.19	0.03	0.24	0.13, 0.26	<0.01
Informational disclosure with online friends	0.13	0.02	0.27	0.10, 0.16	<0.01	0.12	0.02	0.27	0.08, 0.17	<0.01	0.14	0.02	0.25	0.10, 0.19	<0.01

^†^ Controlled for sex, age, BMI, religion, exercise per week, money can be spent each month, have a boy/girl friend, and living place; ^††^ Controlled for age, BMI, religion, exercise per week, money can be spent each month, have a boy/girl friend, and living place; B: unstandardized regression coefficient; SE: standard error; CI: confidence interval; RIIS: Real Interpersonal Interaction Scale; IIIS: Internet Interpersonal Interaction Scale; IGD9-SF: Internet Gaming Disorder Scale. * *p* < 0.05.

## Data Availability

The datasets used and/or analyzed during the current study are available from the corresponding author on reasonable request.
